# Evaluation of factors associated with severe and frequent back pain in high school athletes

**DOI:** 10.1371/journal.pone.0171978

**Published:** 2017-02-21

**Authors:** Matias Noll, Erika Aparecida Silveira, Ivan Silveira de Avelar

**Affiliations:** 1 Postgraduate Program in Health Sciences, Faculty of Medicine, Federal University of Goiás, Goiás, Brazil; 2 Instituto Federal Goiano, Goiás, Brazil; Nanyang Technological University, SINGAPORE

## Abstract

Several studies have shown that half of all young athletes experience back pain (BP). However, high intensity and frequency of BP may be harmful, and the factors associated with BP severity have not been investigated in detail. Here, we investigated the factors associated with a high intensity and high frequency of BP in high school athletes. We included 251 athletes (173 boys and 78 girls [14–20 years old]) in this cross-sectional study. The dependent variables were a high frequency and high intensity of BP, and the independent variables were demographic, socioeconomic, psychosocial, hereditary, anthropometric, behavioural, and postural factors and the level of exercise. The effect measure is presented as prevalence ratio (PR) with 95% confidence interval (CI). Of 251 athletes, 104 reported BP; thus, only these athletes were included in the present analysis. Results of multivariable analysis showed an association between high BP intensity and time spent using a computer (PR: 1.15, CI: 1.01–1.33), posture while writing (PR: 1.41, CI: 1.27–1.58), and posture while using a computer (PR: 1.39, CI: 1.26–1.54). Multivariable analysis also revealed an association of high BP frequency with studying in bed (PR: 1.19, CI: 1.01–1.40) and the method of carrying a backpack (PR: 1.19, CI: 1.01–1.40). In conclusion, we found that behavioural and postural factors are associated with a high intensity and frequency of BP. To the best of our knowledge, this study is the first to compare different intensities and frequencies of BP, and our results may help physicians and coaches to better understand BP in high school athletes.

## Introduction

Back pain (BP) is a common complaint among young athletes, and it has been investigated in several countries including Germany [1], Brazil [2], Iran [3], Canada [4], and Tunisia [5]. The prevalence of BP in young athletes is about 40–50% [6,7] and is similar in developed and developing countries. BP can harm an athlete’s health, as it is associated with increased risk of chronic disease in the spine, sleep disorders, difficulty performing activities of daily living, decreased mobility and quality of life, and consequently reduced performance levels [3,8,9]. BP may also limit participation in training and competitions [6,10].

Researchers have focused on the relationship between sports variables (e.g. the type of sport and frequency and intensity of exercise) and BP [1,3–5,11]. Rossi et al [12] evaluated adolescents belonging to Finnish athletic clubs and verified that 25.9% of boys sought medical assistance for BP. Muller et al [11] reported that the greatest prevalence of BP was found in combat sport athletes. In addition to sports variables, our previous study [2] on high school athletes also evaluated characteristics unrelated to athletic training and found that non-exercise-related variables, and particularly psychosocial, behavioural, and postural variables that are often overlooked by researchers, were strongly related to BP [2]. These findings demonstrated the importance of activities outside the context of sports on an athlete’s health and performance.

The aforementioned studies compared the BP prevalence between athletes with and without BP [1–5,11]. However, they did not consider the degree and frequency of BP; thus, among athletes with BP, those with very low-intensity and/or infrequent pain were grouped together with those who experienced high-intensity and/or frequent pain. Going forward, these critical aspects of BP should be considered through improved analysis methods. This issue is problematic because, first, high-intensity and high-frequency BP is more harmful and should be given more attention [6,9,11,13], and second, in many cases, athletes experience chronic pain during sports [1], and thus the coach and health team must determine ways to reduce BP as much as possible [7,14,15]. Consequently, there is a need to understand the factors associated with severe BP in order to enable health teams to perform more targeted and effective health interventions, given that BP may lead to a high absence rate from training sessions and competitions [3] and affect athletes’ performance [9,16].

To the best of our knowledge, this study is the first to compare different intensities and frequencies of BP in young athletes. The present study was based on the following research questions: a) Is there a relationship between sports variables and high intensity and high frequency BP? b) Which variables explain high intensity and high frequency BP better: exercise or non-exercise variables? To address these questions, we aimed to verify the factors associated with a high intensity and high frequency of BP in high school athletes. We hypothesized that postural- and exercise-related variables (such as weekly frequency, sport modality, and competition) were factors associated with severe and frequent BP.

## Methods

The analyses of the present study were based on data from the Brazilian High School Athlete Study: Health-related Outcomes, a cross-sectional research study performed during the state phase of the 2015 Federal Institutes Games (FIGs) in Brazil [2]. The annual FIGs include both regional and national phases, for which the top-ranked athletes are selected following the state phase.

### Study sample

Students from 14 cities in the state of Goiás, in the Brazilian Midwest, who regularly practiced sports were enrolled as athletes in the Federal Institutes. We invited 320 high school athletes who participated in the FIG in the state of Goiás to take part in this study. The students studied at high schools associated with a technical course, totalling on average 34 hours of class per week. Inclusion criteria were athletes 14–20 years old, with no previous history of musculoskeletal surgery, who participated in one of the following sports activities: volleyball, basketball, handball, or soccer. Among these 320 athletes, 27 were injured, and 42 declined to participate. Thus, 251 athletes (173 [68.9%] boys and 78 [31.1%] girls) were enrolled in our study (S1 Table).

The athletes and their guardians, in the case of minors, voluntarily signed an informed consent form specifically for this study. The present study was approved by the Ethics Committee for Human Research of the Federal University of Goiás.

### Data collection

The Back Pain and Body Posture Evaluation Instrument (BackPEI) self-administered questionnaire was used to determine the prevalence of BP in the previous 3 months [17]. BP intensity (BPI) was evaluated using a visual analogue scale (VAS). The VAS is a continuous scale comprised of a horizontal line 10 centimetres long anchored by no pain (score of 0) and worst imaginable pain (score of 10) [18]. Intensity was classified as follows: mild, 0–3.4; moderate, >3.4–7.4; and severe, >7.4–10 [19]. The BackPEI questionnaire also assessed demographic, socioeconomic, behavioural, and postural factors and the level of exercise.

Athletes with BP were stratified according to BPI into low intensity (mild intensity) and high intensity (moderate and severe intensities) groups. Additionally, they are stratified according to BP frequency (BPF) into low frequency (BP once per month or less) and high frequency (BP once per week or more) groups.

The Brazilian National School-Based Health Survey (PeNSE) self-administered questionnaire [20,21] was used to assess behavioural (“Did you smoke frequently last month?”; “Did you consume alcohol frequently last month?”; answers: “yes” or “no”), and psychosocial (“How frequently did you feel lonely last year?”; “How frequently did you lose sleep last year due to something that concerned you?”; “How frequently did you feel intimidated last month?”; answers: “never”, “rarely”, “sometimes”, or “almost every day”) factors. Psychosocial results were grouped into “never and rarely” and “sometimes or more”. The PeNSE focuses on assessing risk and protective factors for health among students enrolled in public and private schools in Brazil [22].

Although the BackPEI and PeNSE have been widely used in the literature, we evaluated their reliability in 34 high school students who did not participate in the study. A test-retest protocol with a 7-day interval was used, and good and very good values [23] for all BackPEI (κ range: 0.704–0.944) and PeNSE questions (κ range: 0.701–0.841) were verified.

Each athlete’s body mass and height were measured, and the body mass index (BMI) (kg/m^2^) was calculated. Students wore light clothes (swimwear) at the time of data collection and were instructed to remain standing upright, with the face directed forward and shoulders relaxed. To measure body weight and height, a digital scale with maximum capacity of 150 kg and accuracy of 100 g (Plenna-MEA-03140, São Paulo, Brazil) and stadiometer accurate to 0.1 mm were used, respectively. Athletes were classified according to the World Health Organization Growth Reference standard [24]. The standard deviation (SD) of BMI Z-score was used: normal weight (−2 SD < BMI Z-score < 1 SD), overweight (1 SD ≤ BMI Z-score < 2 SD), or obese (BMI Z-score ≥ 2 SD).

We evaluated handgrip and lumbar strength using a hand dynamometer (EMG System, model TRF_MAN200, São José dos Campos, Brazil) and a lumbar dynamometer (EMG System, model TRF_ELMB200). Both dynamometers (nominal capacity 200 kg, sensitivity 2 mV/V ± 10%, error < 0.03%) were calibrated before data collection and adjusted according to each athlete’s size. Handgrip strength were collected with the athlete seated with the elbow flexed at 90°, shoulders adducted and neutrally rotated, forearm in the neutral position, and wrist in 10° extension [25,26]. Each athlete performed two valid isometric trials lasting 5 s to measure lumbar and right and left hand strength, and the larger value was recorded. The average handgrip strength of both hands was calculated [27]. A rest period of 60 s between trials was permitted. All values were normalized by body weight. Participants were encouraged to achieve maximum handgrip strength. More details concerning test-retest reliability were reported in our previous study [2].

Weight distribution was evaluated using two force plates (AMTI, Dual-Top Accusway force plate, Watertown, MA) with 6-channel digital output, capacity of 1112 N/136 Nm, sensitivity of 0.67 μV/VN, natural frequency of 120 Hz. All participants stood quietly with feet shoulder width apart, their arms relaxed at their sides, throughout the duration of testing [28]. Weight distribution was assessed while the athlete stood for 30 s with one foot on each force plate and looked at a fixed point. The first 10 s of measurement were discarded, and the average weight under each foot was calculated for the remaining 20 s.

The weight asymmetry index was calculated in accordance with the method used in our previous study [2]. The asymmetry index score represents the difference in weight-bearing between sides. Perfect symmetry (50% weight-bearing on each limb) during standing is considered ideal [28,29]. The data for handgrip and lumbar strength and weight asymmetry were divided into two groups according to the median. We designated the group with results below the median as ‘low’, and that with results above the median as ‘high’.

### Statistical analysis

Data were analysed using descriptive and inferential statistics. First, handgrip and lumbar strength were compared between the low and high BPI groups and the low and high BPF groups. Because the lumbar strength data are parametric and handgrip strength data are non-parametric, the independent test t and the Mann-Whitney U test were used, respectively, for these comparisons. Second, we performed the Wald chi-square test to assess the association of each outcome (BPI and BPF) with the independent variables. The demographic, socioeconomic, psychosocial, anthropometric, behavioural, and postural factors and level of exercise were considered independent variables. Independent variables with a significance level of p < 0.20 in bivariate analysis were included in a Poisson regression model with robust variance, and the assumptions required for Poisson regression to yield a valid result were respected [30]. The effect measure is presented as prevalence ratio (PR) with 95% confidence interval (CI). The threshold α = 0.05 was used to indicate statistical significance.

## Results

Of 251 athletes, 104 (41.3%) reported experiencing BP in the previous 3 months (age 16.5 ± 1.3 years, body mass 66.3 ± 12.8 kg, height 1.69 ± 0.09 m) and were included in the present analysis (Table 1).

**Table 1:** Frequency and percentage of high school athletes categorized according to sex and age.

Age	Male	Female	Total
	n (%)	n (%)	n (%)
14–16 years	29 (48.3)	29 (65.9)	58 (55.8)
17–20 years	31 (51.7)	15 (34.1)	46 (44.2)
Total	60 (57.7)	44 (42.3)	104 (100)

**Fig 1:**
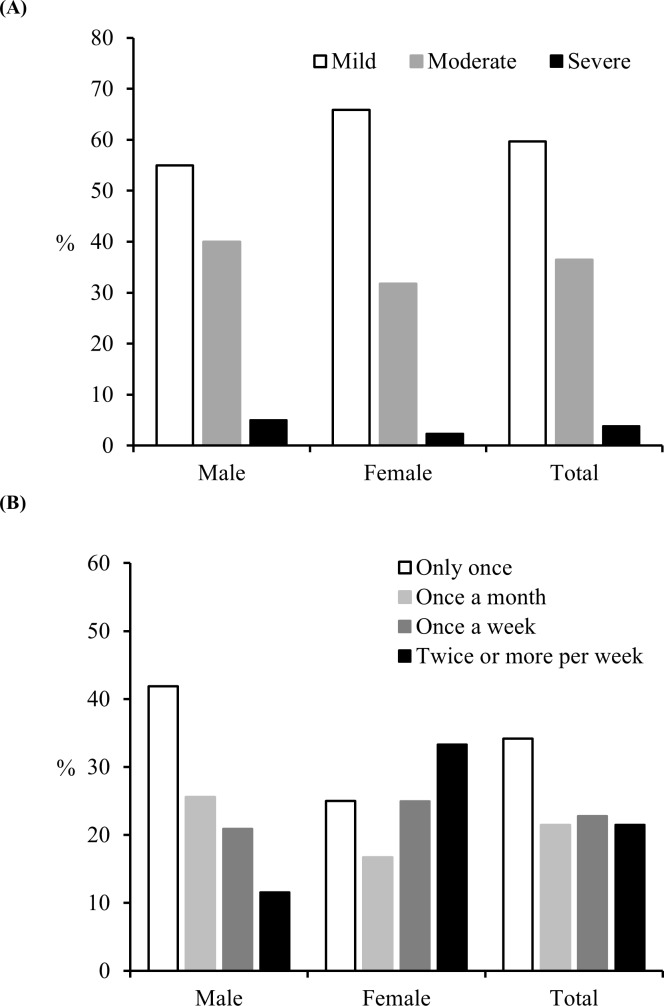
Prevalence of back pain intensity (A) and back pain frequency (B) in the previous 3 months.

The results indicated that 59.6% of athletes had mild-intensity BP (Fig 1A). The BP frequency was uncertain in one-fourth of the athletes (n = 25), and these athletes were excluded from all analyses. The prevalence of BP one or more times per week was substantial (44.3%) (Fig 1B). Of 251 athletes, 7.6% experienced BP 4 or more times per week.

**Fig 2:**
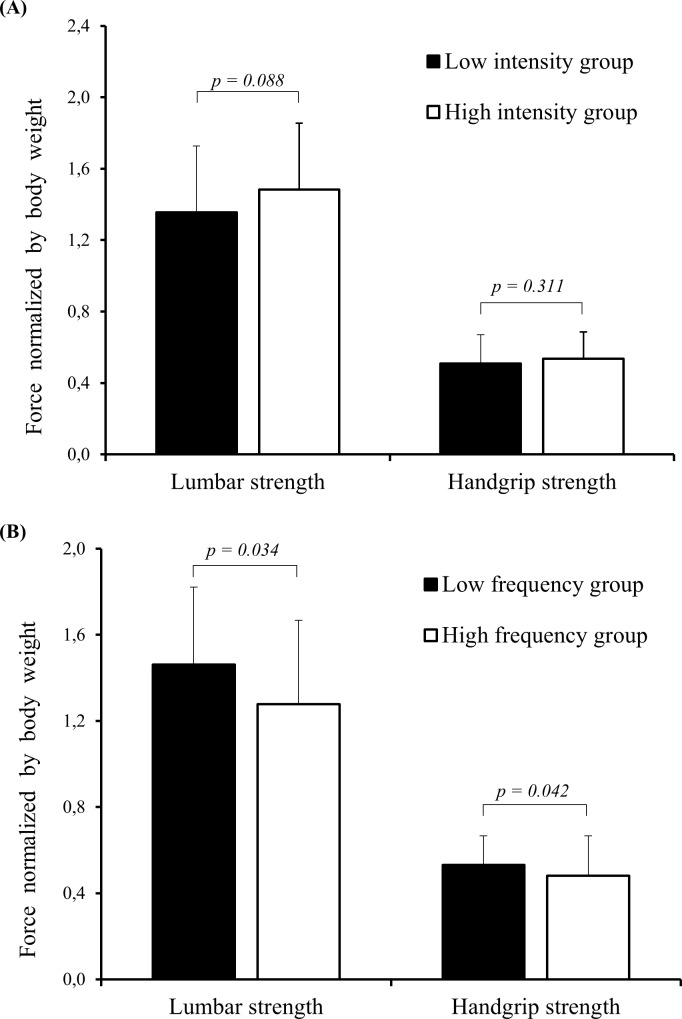
**Handgrip and lumbar strength stratified according to back pain intensity (A) and back pain frequency (B).** Independent t test was used for lumbar strength analysis. Mann-Whitney U test was used for handgrip strength analysis.

Fig 2 shows significant differences in handgrip and lumbar strength between the low frequency and high frequency groups. The high BPF group had lower handgrip (U = 564, p = 0.04) and lumbar strength [t(77) = 2.15, p = 0.03] than the low BPF group. BPI did not differ between the low intensity and high intensity groups.

Bivariate analysis revealed an association of BPI with behavioural and postural variables, and BPF with sex, exercise level, and postural variables (Tables 2–5). After performing multivariate analysis, only the time spent using a computer and sitting in a posture to write and use a computer remained associated with BPI, and reading or studying in bed and the method of carrying a backpack remained associated with BPF (Table 6). These behavioural and postural factors were associated with increasing BPF and BPI. Athletes who used a non-recommended sitting posture to write (RP, 1.41; 95%CI, 1.27–1.58) and use computer (RP, 1.39; 95%CI 1.26–1.54) demonstrated higher prevalence ratios and were more predisposed to greater BPI.

**Table 2:** Association (χ^2^) and prevalence ratio of back pain intensity with independent variables (demographic, socioeconomic, anthropometric, and psychosocial).

Independent variable	n (%)	Prevalence of high-intensity BP (%)	Prevalence ratio (95% CI)	*p* ^a^
**Demographic**				
*Sex (n = 104)*				*0*.*259*
Male	60 (57.7)	27 (45)	1	
Female	44 (42.3)	15 (34.1)	0.92 (0.81–1.06)	
*Age (n = 104)*				*0*.*865*
14–16 years	58 (55.8)	23 (39.7)	1	
17–20 years	46 (44.2)	19 (41.3)	1.01 (0.88–1.16)	
*Skin colour (n = 103)*				*0*.*639*
Black	15 (14.6)	4 (26.7)	1	
White	29 (28.2)	11(37.9)	1.09 (0.87–1.35)	
Brown	43 (41.7)	19 (44.2)	1.14 (0.93–1.40)	
Other (Asian and Indigenous)	16 (15.6)	7 (43.8)	1.13 (0.89–1.45)	
**Socioeconomic**				
*Work (n = 103)*				*0*.*437*
No	91 (88.3)	35 (38.5)	1	
Yes	12 (11.7)	6 (50)	1.08 (0.88–1.33)	
*Maternal education (n = 100)*				*0*.*430*
Basic education	24 (24)	11 (45.8)	1	
High school and college	76 (76)	28 (36.8)	0.94 (0.80–1.10)	
*Paternal education (n = 94)*				*0*.*586*
Basic education	45 (47.9)	19 (42.2)	1	
High school and college	49 (52.1)	18 (36.7)	0.96 (0.83–1.11)	
**Anthropometric**				
*Body mass index (n = 104)*				*0*.*885*
Normal weight	70 (67.3)	28 (40)	1	
Overweight	28 (26.9)	14 (41.2)	0.98 (0.80–1.21)	
*Weight asymmetry index (n = 88)*				*0*.*516*
Low asymmetry	44 (50)	17 (38.6)	1	
High asymmetry	44 (50)	20 (45.5)	1.05 (0.91–1.21)	
**Psychosocial**				
*Feeling lonely last year (n = 100)*				*0*.*997*
Never and rarely	61 (61)	25 (41)	1	
Sometimes or more	39 (39)	16 (41)	1 (0.87–1.15)	
*Loss of sleep last year (n = 99)*				*0*.*643*
Never and rarely	63 (63.3)	25 (39.7)	1	
Sometimes or more	36 (36.4)	16 (44.4)	1.03 (0.90–1.19)	
*Feeling intimidated last month (n = 102)*				*0*.*992*
Never and rarely	79 (77.5)	31 (39.2)	1	
Sometimes or more	23 (22.5)	9 (39.1)	1 (0.85–1.18)	

^a^ Bivariate analysis. Wald chi-squared test.

^b^ Significant association (*p* < 0.05).

**Table 3:** Association (χ^2^) and prevalence ratio of back pain intensity with independent variables (exercise level, behavioural, and postural).

Independent variable	n (%)	Prevalence of high-intensity BP (%)	Prevalence ratio (95% CI)	*p*^a^
**Exercise level**				
*Physical exercise weekly frequency (n = 96)*				*0*.*062*
1–2 days	47 (49)	23 (48.9)	1	
3 or more days per week	49 (51)	15 (30.6)	0.88 (0.76–1.01)	
*Sport modality (n = 104)*				*0*.*512*
Handball	21 (20.2)	7 (33.3)	1	
Soccer	42 (40.4)	15 (35.7)	1.02 (0.85–1.22)	
Basketball	11 (10.6)	6 (54.5)	1.16 (0.91–1.48)	
Volleyball	30 (28.8)	14 (46.7)	1.10 (0.91–1.34)	
*Competition (n = 99)*				*0*.*561*
1 per year	53 (53.5)	20 (37.7)	1	
2 or more per year	46 (45.5)	20 (43.5)	1.04 (0.91–1.19)	
*Handgrip strength (n = 104)*				*0*.*206*
Low	52 (50)	17 (32.7)	1	
High	52 (50)	25 (48.1)	1.11 (0.97–1.27)	
*Lumbar strength (n = 104)*				*0*.*106*
Low	52 (50)	17 (32.7)	1	
High	52 (50)	25 (48.1)	1.11 (0.97–1.27)	
**Behavioural**				
*Time spent watching television per day (n = 95)*				*0*.*908*
0–1 hours	59 (62.1)	22 (37.3)	1	
2 or more hours	36 (37.9)	13 (36.1)	0.99 (0.86–1.15)	
*Time spent using a computer per day (n = 88)*				*0*.*049* ^b^
0–1 hours	36 (40.9)	10 (27.8)	1	
2 or more hours	52 (59.1)	25 (48.1)	1.16 (1.01–1.34)	
*Time sleeping per night (n = 103)*				*0*.*051*
0–6 hours	42 (40.8)	21 (50)	1.35 (1.05–1.74)	
7 hours	36 (35)	12 (33.3)	1.14 (0.88–1.47)	
8–9 hours (Recommended)	15 (14.6)	3 (20)	1	
10 hours	10 (9.7)	6 (60)	1.79 (1.03–2.15)	
*Reading/studying in bed (n = 104)*				*0*.*638*
No	17 (16.3)	6 (35.3)	1	
Yes	87 (83.7)	44 (50.6)	1.05 (0.87–1.26)	
*Smoking habits last month (n = 104)*				*0*.*648*
No	95 (91.3)	46 (48.4)	1	
Yes	9 (8.7)	4 (44.4)	0.94 (0.74–1.20)	
*Alcohol consumption last month (n = 104)*				*0*.*605*
No	65 (62.5)	31 (47.7)	1	
Yes	39 (37.5)	19 (48.7)	1.04 (0.90–1.19)	
**Postural**				
*Sleeping posture (n = 90)*				*0*.*369*
Supine and lateral decubitus	46 (51.1)	24 (52.2)	1	
Prone	44 (48.9)	21 (47.7)	0.94 (0.81–1.08)	
*Posture adopted to lift object from floor (n = 104)*				*0*.*317*
Recommended	14 (13.5)	6 (42.9)	1	
Not recommended	90 (86.5)	44 (48.9)	1.11 (0.91–1.35)	
*Method of carrying a backpack (n = 104)*				*0*.*313*
Recommended (both shoulders)	71 (68.3)	35 (49.3)	1	
Not recommended	33 (31.7)	15 (45.5)	0.93 (0.80–1.07)	
*Sitting posture to write (n = 104)*				*<0*.*001* ^b^
Recommended	3 (2.9)	0 (0)	1	
Not recommended	101 (97.1)	42 (41.6)	1.42 (1.32–1.51)	
*Sitting posture on a bench (n = 104)*				*0*.*798*
Recommended	3 (2.9)	1 (33.3)	1	
Not recommended	101 (97.1)	41 (40.6)	1.05 (0.70–1.58)	
*Sitting posture to use a computer (n = 104)*				*<0*.*001* ^b^
Recommended	8 (7.7)	0 (0)	1	
Not recommended	96 (92.3)	42 (41.6)	1.44 (1.34–1.54)	

^a^ Bivariate analysis. Wald chi-squared test.

^b^ Significant association (*p* < 0.05).

**Table 4:** Association (χ^2^) and prevalence ratio of back pain frequency with independent variables (demographic, socioeconomic, anthropometric, and psychosocial).

Independent variable	n (%)	Prevalence of high-frequency BP(%)	Prevalence ratio (95% CI)	*p* ^a^
**Demographic**				
*Sex (n = 79)*				*0*.*018* ^b^
Male	43 (54.4)	14 (32.6)	1	
Female	36 (45.6)	21 (58.3)	1.19 (1.03–1.38)	
*Age (n = 79)*				*0*.*626*
14–16 years	45 (57)	21 (46.7)	1	
17–20 years	34 (43)	14 (41.2)	0.96 (0.82–1.12)	
*Skin colour (n = 79)*				*0*.*724*
Black	15 (19)	6 (40)	1	
White	24 (30.4)	12 (50)	1.12 (0.88–1.43)	
Brown	31 (39.2)	12 (38.7)	1.01 (0.81–1.25)	
Other (Asian and Indigenous)	9 (11.4)	5 (55.6)	1.08 (0.90–1.30)	
**Socioeconomic**				
*Work (n = 79)*				*0*.*477*
No	70 (88.6)	32 (45.7)	1	
Yes	9 (11.4)	3 (33.3)	0.92 (0.72–1.17)	
*Maternal education (n = 78)*				*0*.*701*
Basic education	19 (24.4)	9 (47.4)	1	
High school and College	59 (75.6)	25 (42.4)	0.97 (0.81–1.15)	
*Paternal education (n = 73)*				*0*.*322*
Basic education	33 (45.2)	17 (51.4)	1	
High school and College	40 (54.8)	16 (40)	0.92 (0.79–1.08)	
**Anthropometric**				
*Body mass index (n = 79)*				*0*.*986*
Normal weight	52 (65.8)	23 (44.2)	1	
Overweight	27 (34.2)	12 (44.4)	1.01 (0.859–1.17)	
*Weight asymmetry index (n = 67)*				*0*.*842*
Low asymmetry	36 (53.7)	17 (47.2)	1	
High asymmetry	31 (46.3)	12 (38.7)	0.94 (0.79–1.11)	
**Psychosocial**				
*Feeling lonely last year (n = 78)*				*0*.*641*
Never and rarely	49 (62.8)	21 (42.9)	1	
Sometimes or more	29 (37.2)	14 (48.3)	1.03 (0.89–1.21)	
*Loss of sleep last year (n = 77)*				*0*.*115*
Never and rarely	53 (68.8)	21 (39.6)	1	
Sometimes or more	24 (31.2)	14 (58.3)	1.13 (0.97–1.33)	
*Feeling intimidated last month (n = 78)*				*0*.*934*
Never and rarely	60 (76.9)	26 (43.3)	1	
Sometimes or more	18 (23.1)	8 (44.4)	1.01 (0.84–1.21)	

^a^ Bivariate analysis. Wald chi-squared test.

^b^ Significant association (*p* < 0.05).

**Table 5:** Association (χ^2^) and prevalence ratio of back pain frequency with independent variables (exercise level, behavioural, and postural).

Independent variable	n (%)	Prevalence of high-frequency BP (%)	Prevalence ratio (95% CI)	*p* ^a^
**Exercise level**				
*Physical exercise weekly frequency (n = 73)*				*0*.*138*
1–2 days	35 (47.9)	18 (51.4)	1	
3 or more days per week	38 (52.1)	13 (34.2)	0.88 (0.76–1.04)	
*Sport modality (n = 79)*				*0*.*339*
Handball	15 (19)	8 (53.3)	1	
Soccer	33 (41.8)	14 (42.3)	0.93 (0.76–1.13)	
Basketball	6 (7.6)	1 (16.7)	0.76 (0.56–1.03)	
Volleyball	25 (31.6)	12 (48)	0.96 (0.78–1.19)	
*Competition (n = 76)*				*0*.*053*
1 per year	40 (52.6)	22 (55)	1	
2 or more per year	36 (47.4)	12 (33.3)	0.86 (0.74–1.01)	
*Handgrip strength (n = 79)*				*0*.*087*
Low	39 (49.4)	21 (53.8)	1	
High	40 (50.6)	14 (35)	0.88 (0.76–1.02)	
*Lumbar strength (n = 79)*				*0*.*048* ^b^
Low	40 (50.6)	22 (55)	1	
High	39 (49.4)	13 (33.3)	0.86 (0.74–0.99)	
**Behavioural**				
*Time spent watching television per day (n = 77)*				*0*.*565*
0–1 hours	51 (66.2)	22 (43.1)	1	
2 or more hours	26 (33.8)	13 (50)	1.05 (0.89–1.23)	
*Time spent using a computer per day (n = 71)*				*0*.*839*
0–1 hours	32 (45.1)	14 (43.8)	1	
2 or more hours	39 (54.9)	18 (46.2)	1.02 (0.87–1.19)	
*Time sleeping per night (n = 78)*				*0*.*859*
0–6 hours	38 (48.7)	16 (42.1)	1.40 (1.13–1.74)	
7 hours	23 (29.5)	11 (47.8)	1.06 (0.82–1.36)	
8–9 hours (Recommended)	10 (12.8)	4 (40)	1	
10 hours	7 (9)	4 (57.1)	1.12 (0.82–1.54)	
*Reading/studying in bed (n = 79)*				*0*.*111*
No	15 (19)	4 (26.7)	1	
Yes	64 (81)	31 (48.4)	1.17 (0.96–1.42)	
*Smoking habits last month (n = 79)*				*0*.*936*
No	72 (91.1)	32 (44.4)	1	
Yes	7 (8.9)	3 (42.9)	0.99 (0.76–0.29)	
*Alcohol consumption last month (n = 79)*				*0*.*587*
No	50 (63.3)	21 (42)	1	
Yes	29 (36.7)	14 (48.3)	1.04 (0.89–1.22)	
**Postural**				
*Sleeping posture (n = 69)*				*0*.*704*
Supine and lateral decubitus	34 (49.3)	14 (41.2)	1	
Prone	35 (50.7)	16 (45.7)	1.03 (0.88–1.22)	
*Posture adopted to lift object from floor (n = 79)*				*0*.*401*
Recommended	12 (15.2)	4 (33.3)	1	
Not recommended	67 (84.8)	31 (46.3)	1.10 (0.88–1.36)	
*Method of carrying a backpack (n = 79)*				*<0*.*001* ^b^
Recommended (both shoulders)	53 (67.1)	16 (30.2)	1	
Not recommended	26 (32.9)	19 (73.1)	1.33 (1.16–1.52)	
*Sitting posture to write (n = 79)*				*<0*.*001* ^b^
Recommended	2 (2.5)	0 (0)	1	
Not recommended	77 (97.5)	35 (45.5)	1.45 (1.35–1.57)	
*Sitting posture on a bench (n = 79)*				*0*.*868*
Recommended	2 (2.5)	1 (50)	1	
Not recommended	77 (97.5)	34 (44.2)	0.96 (0.60–1.53)	
*Sitting posture to use a computer (n = 79)*				*0*.*682*
Recommended	8 (10.1)	3 (37.5)	1	
Not recommended	71 (89.9)	32 (45.1)	1.05 (0.82–1.36)	

^a^ Bivariate analysis. Wald chi-squared test.

^b^ Significant association (*p* < 0.05).

**Table 6:** Results of multivariate analysis and adjusted prevalence ratio for a high intensity and high frequency of back pain and independent variables.

Independent variable	Adjusted prevalence ratio (95% CI)	*p* ^a^
**High intensity**		
*Physical exercise weekly frequency*		*0*.*059*
1–2 days	1	
3 or more days per week	0.88 (0.77–1.01)	
*Lumbar strength*		*0*.*059*
Low	1	
High	1.13 (0.99–1.28)	
*Time spent using a computer per day*		*0*.*044* ^b^
0–1 hours	1	
2 or more hours	1.15 (1.01–1.33)	
*Time sleeping per night*		*0*.*067*
0–6 hours	1.18 (0.97–1.43)	
7 hours	1.03 (0.83–1.26)	
8–9 hours (Recommended)	1	
10 hours	1.27 (0.99–1.63)	
*Sitting posture to write*		*<0*.*001* ^b^
Recommended	1	
Not recommended	1.41 (1.27–1.58)	
*Sitting posture to use a computer*		*<0*.*001* ^b^
Recommended	1	
Not recommended	1.39 (1.26–1.54)	
**High frequency**		
*Sex*		*0*.*329*
Male	1	
Female	1.12 (0.89–1.41)	
*Loss of sleep last year*		*0*.*125*
Never and rarely	1	
Sometimes or more	1.13 (0.97–1.31)	
*Physical exercise weekly frequency*		*0*.*117*
1–2 days	1	
3 or more days per week	0.88 (0.74–1.03)	
*Competition*		*0*.*163*
1 per year	1	
2 or more per year	0.90(0.78–1.04)	
*Handgrip strength*		*0*.*866*
Low	1	
High	1.02 (0.81–1.28)	
*Lumbar strength*		*0*.*304*
Low	1	
High	0.90 (0.74–1.09)	
*Reading/studying in bed*		*0*.*039* ^b^
No	1	
Yes	1.19 (1.01–1.40)	
*Method of carrying a backpack*		*<0*.*001* ^b^
Recommended (both shoulders)	1	
Not recommended	1.30 (1.13–1.49)	
*Sitting posture to write*		*0*.*051*
Recommended	1	
Not recommended	1.27 (0.99–1.60)	

^a^ Multivariate analysis.

^b^ Significant association (*p* < 0.05).

## Discussion

In our study, the results of multivariate analysis indicated no association between sports characteristics and BPI or BPF. However, we found that high BPI and BPF were prevalent in athletes with poor behavioural and postural habits. These results are interesting, because usually only sports characteristics, not activities of daily living, have been considered a detriment to athletes’ BP.

The present study revealed a relationship between BPI and time spent using a computer. No previous studies have assessed the relationship between BPI and time spent using a computer in young athletes. In a study of 14–18-year-old students, Halaka et al [31] reported that frequent computer-related activities are a risk factor for neck, shoulder, and low back pain. Studies of college students demonstrated that periods in a sustained sitting posture and increased back flexion from sitting are significantly associated with BP [32]. Additionally, studies of workers revealed a positive association between the time spent sitting and BP [33]. Long-term sitting increases compression on the intervertebral discs, which leads to disc malnutrition, and it may compromise the integrity of the musculoskeletal system [34].

Similarly, the problems associated with the sitting position can worsen if one’s posture remains poor. We found an association between a high BPI and sitting posture while writing and using a computer. Students who remained seated for long periods throughout the day in an inappropriate posture (e.g., an unaligned head position, hyperlordotic or slumped trunk, and unaligned shoulders) [35,36], are predisposed to fatigue and higher levels of pain [37,38]. Likewise, a recent study [39] that examined 59 students using panoramic spinal radiographs showed a strong association between inadequate posture and increased thoracic kyphosis. However, it is important to note that the definition of an ideal body posture is complex and involves several variables including biomechanical, neuromuscular, and psychosocial factors [40–42]. Therefore, we used the BackPEI questionnaire to evaluate posture by figures specifically for each sex, and we defined the recommended posture as a neutral position of the spine [36,37], which is widely accepted as a vital position for a lower risk for BP [34] and proper body function [32,43].

Multivariate analysis also indicated an association between high BPF and reading or studying in bed and method of carrying a backpack. To read or study in bed, students lay in an awkward posture. A non-neutral lying posture can lead to improper bending of the intervertebral disks, which may cause damage to spine structures in the long-term [44]. Asymmetrical carrying of a backpack can have a similar effect.

As has been widely reported in children and adolescents [45,46], athletes who do not carry backpacks evenly on both shoulders presented a high prevalence of BP. Supporting a backpack unevenly across the shoulders may increase the sagittal angles and loads on the spine, causing a compensation mechanism to reposition the load over the subject’s centre of mass, i.e. elevate the loaded shoulder and trunk (lateral flexion) away from the load [47]. Furthermore, Neuschwander et al [48] and Muslim et al [49] reported that backpack loads increase compression of the lumbar disc and the intensity of BP. Although these factors are not generally considered in research or by coaches during sports practice, they play a significant role in athletic function in the context of injury or illness [50].

The relationship between BP and posture can be considered a functional problem; thus, it is likely transient and reversible [51]. However, if spinal structures are exposed to prolonged and repetitive mechanical loading, disc degeneration and tissue injuries can occur, and consequently the problem can become chronic and structural [6,34,51]. This perspective highlights the importance of health education programs, such as Back Schools, good quality and sufficient duration of sleep, maintenance of neutral posture throughout sporting activities as well in non-exercise-related activities at school and at home, and enhanced thoracic spine mobility [52].

Although we evaluated many variables, no association of BPI and BPF with demographic, socioeconomic, anthropometric, and psychosocial factors or the exercise level was found. Although sleep time was not associated with BP, insufficient sleep may impair athletes’ musculoskeletal tissue recovery and make them more tired, contributing to the adoption of inappropriate postures [53–55]. Although we found no association of BPI or BPF with weight distribution, this variable has received relatively little attention, especially in the context of exercise, and due to the importance of symmetry in sports activities, we believe that it warrants continued assessment [28,29]. The absence of an observed association between BP and exercise level may be the result of our study being limited to similar sports activities. For example, Muller et al [11] demonstrated that athletes in game sports had a lower risk of BP, whereas athletes in combat sports (such as boxing and judo) had the highest prevalence of BP.

The characteristics of a sport may also predispose athletes to particular risks [1,9]; thus, it may be beneficial in future studies to assess the competitive season or training cycle [7]. Furthermore, our study did not investigate other possible explanatory risk factors besides flexibility levels, abdominal strength, postural alterations, muscle asymmetries, overtraining, and recovery [15]. Thus, these variables should be evaluated in future research.

The present study has several limitations. First, the cross-sectional study design does not permit inferences into cause and effect. Thus, we stress the need for future longitudinal studies to evaluate BP outcomes in more detail. Second, assessment by self-reported questionnaires should be interpreted with caution, because memory bias may be present, which can lead to either overestimation or underestimation in the responses. To address this concern, we investigated the reliability of the BackPEI and PeNSE questionnaires using a test-retest protocol, which indicated good and very good reliability, respectively.

Our study is the first to evaluate a number of variables beyond sports-related factors in high school athletes and to compare different intensities and frequencies of BP. This approach is similar to that proposed by Puentedura and Louw [50] to evaluate BP from a more complex biopsychosocial viewpoint, extending beyond biological factors to include psychological, behavioural, social, sociodemographic, and economic variables. Studies that evaluate multiple factors related to BP are essential, given the recent evidence showing that this condition, along with poor health behaviours, are commonly retained in adulthood [6,34,37,56]. Recent prospective studies [1,57] with athletes observed similar trends.

Our results suggest that more attention be paid to education and rehabilitation using a structured program that focuses on athletes in general and evaluates specific motions, behaviours of daily living, postures, and activities required in the performance of the athlete’s chosen sport [7,15]. Therefore, we recommend the establishment of multidisciplinary health teams within athletic clubs to prevent unilateral development and muscle deficits, training beyond the specific demands of the target sport, reduction of sedentary behaviour, and identification of psychosocial barriers to promote education programs with athletes as well parents. These issues must be addressed as an integral part of training in order to optimize both health outcomes and sports performance.

In conclusion, BPI and BPF are associated with behavioural and postural habits. This study adds to the current knowledge about BP in athletes, and it has identified some novel factors affecting BP that should be carefully considered by the people responsible for the sporting life of athletes.

## Supporting information

S1 Table Datasets of participants. (XLSX)
